# Reconciling opposite conclusions in umbrella species evaluation

**DOI:** 10.1111/cobi.70106

**Published:** 2025-08-11

**Authors:** Tatiane Micheletti, Frances E. C. Stewart, Samuel Hache, Eliot J. B. McIntire

**Affiliations:** ^1^ Faculty of Forestry University of British Columbia Vancouver British Columbia Canada; ^2^ Department of Forest Sciences Technische Universität Dresden Dresden Germany; ^3^ Biology Department Wilfrid Laurier University Waterloo Ontario Canada; ^4^ Canadian Wildlife Service Environment and Climate Change Canada Yellowknife, Northwest Territories Canada; ^5^ Pacific Forestry Centre, Canadian Forest Service Natural Resources Canada Victoria British Columbia Canada

**Keywords:** biodiversity conservation, boreal caribou, conservation efficiency, conservation planning, conservation strategies, rangifer tarandus caribou, umbrella species concept, wildlife management

With accelerating species decline, prioritizing protection of umbrella species is appealing. This strategy assumes that protecting one species confers a “protective umbrella” to co‐occurring ones (Fleishman et al., 2000), improving conservation efficiency. However, no standard criteria exist to quantify the value of an umbrella species. Consequently, evaluations of the same umbrella species may provide opposite conclusions, as boreal woodland caribou (*Rangifer tarandus caribou*) (henceforth caribou) exemplify. With important implications for conservation planning, literature should be carefully reconciled.

**FIGURE 1:**
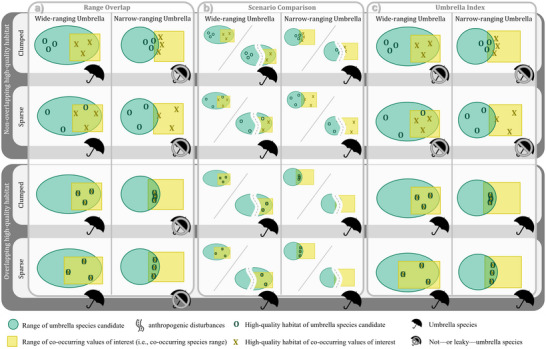
Three commonly used approaches for testing umbrella candidate species: (a) determine overlap of ranges of umbrella species (green circles) and co‐occurring species (yellow squares), (b) compare potential outcomes of conservation of umbrella species’ undisturbed range (i.e., conservation‐oriented scenario) with disturbed range (i.e., road, use‐oriented scenario; diagonal line indicates comparison between scenarios), and (c) determine how much the high‐quality habitat of the umbrella species (letter O) overlaps with that of co‐occurring ones (letter X). Rows represent different habitat configurations (umbrella species’ high‐quality habitat clumped or sparse and overlapping or not with co‐occurring species). Even under the same underlying spatial pattern, these methods (range‐overlap, scenario‐comparison, and umbrella‐index approaches) can yield opposite conclusions regarding umbrella effectiveness.

Over the past decades, caribou populations have significantly declined (Hebblewhite, 2017), prompting legal listings, recovery efforts (Government of Canada, 2021), and research, including quantifying their value as an umbrella species. For example, although Drever et al. (2019) and Labadie et al. (2024) suggest the caribou is a good umbrella species for boreal landbirds, Micheletti et al. (2023) conclude the umbrella may leak. We suggest that this apparent discrepancy likely stems from the different methods used to evaluate umbrella effectiveness—including their spatial scale—rather than different spatial scales or locations alone (Figure 1).

Umbrella species effectiveness is often determined based on whether varying levels of range‐wide protection conferred on one species protect other species (Bichet et al., 2016; Johnson et al., 2022; Labadie et al., 2024; Roberge & Angelstam, 2004). In simple cases, an umbrella species’ value is tested by examining the level of overlap between that species range with other species ranges. A high amount of overlap is interpreted as a high umbrella value (e.g., Nicholson et al., 2013; Roberge & Angelstam, 2004; Figure 1a). In more complex cases, hypothetical umbrella species’ protection and conservation‐oriented management (e.g., low‐intensity forestry) are compared with no‐protection and use‐oriented management (e.g., high‐intensity forestry) scenarios (e.g., Labadie et al., 2024; Figure 1b). Wide‐ranging species are common umbrella candidates (Bichet et al., 2016; Johnson et al., 2022; Labadie et al., 2024; Nicholson et al., 2013) because conservation of large areas—if properly implemented—can increase protection of other species (Roberge & Angelstam, 2004).

The wide range of caribou in Canada's boreal forest overlaps with 90% of all boreal mammals and birds (Drever et al., 2019), covering many hotspots (Johnson et al., 2022) and high‐quality areas for co‐occurring species. Unsurprisingly, traditional analyses of caribou habitat (i.e., approaches based on range overlap and use scenarios) often conclude caribou are an effective umbrella (Bichet et al., 2016; Labadie et al., 2024), akin to other wide‐ranging species (e.g., Nicholson et al., 2013). However, land protection rarely covers a wide‐ranging species’ entire distribution and caribou's high‐quality habitat does not generally overlap with other species’ high‐quality habitat. In the Northwest Territories, for example, caribou's high‐quality habitat (i.e., 250 × 250‐m cell; assessed using resource selection values [DeMars et al., 2020]) overlaps with those of only a small proportion of the boreal landbird community (Micheletti et al., 2023).

When a desired area for conservation is defined (e.g., 30% by 2030 [Eckert et al., 2023]), as opposed to protecting all the land it is possible to protect, it becomes ineffective to assess an umbrella species based on the percentage of range overlap with co‐occurring species or based on a comparison between no‐protection versus protection scenarios. Instead, an umbrella candidate may be more effectively assessed by comparing outcomes of protecting its high‐quality habitat (i.e., primary focus of protection within a species’ range) with outcomes protecting an equivalent area at random locations (i.e., a null model approach [Kerr, 1997]), or other criteria (i.e., an alternative model approach). Determining whether conservation of a proposed umbrella species’ high‐quality habitat would provide more conservation opportunities for other species than expected by chance alone could help promote more effective conservation planning. Such an umbrella index approach can reduce misinterpretation of results and enable more accurate assessments of conservation gains.

In their development and implementation of an umbrella index, Micheletti et al. (2023) found that caribou may not be an effective umbrella for boreal landbirds. Prioritizing caribou needs would be more beneficial than protecting random habitat for <20% of the focal landbird species (*n* = 71). Micheletti et al.’s (2023) results are consistent with the results of multiobjective studies. Martin et al. (2022) observed low overlap between priority areas for caribou conservation and other biodiversity objectives, except carbon stocks. Similarly, Johnson et al. (2022) concluded that, although caribou as an umbrella species could help protect, for example, carbon stocks, this strategy is unlikely to be efficient for achieving multiple conservation targets due to a lack of spatial overlap between caribou and most other values.

With conflicting findings about a species’ umbrella value, understanding the underlying assessment methods can help reconcile these findings. The umbrella index (Micheletti et al., 2023; Figure 1c) provides a potential standardized method, but it could be improved by accounting for habitat size, configuration, and connectivity (Favreau et al., 2006). Micheletti et al.’s (2023) “leaky umbrella” example hinges on a comparison of the candidate umbrella species’ high‐quality habitat with equally sized random areas; altering the comparison criteria could change the results.

Using a widely distributed flagship species as an umbrella species is appealing because such species can provide conservation opportunities for otherwise unprotected areas (Runge et al., 2019). Yet, single‐species strategies rarely meet broad biodiversity targets (Andelman & Fagan, 2000), especially in frameworks aiming to protect specific areas with finite resource allocations. Caribou could serve as an effective umbrella for the boreal ecosystem if large proportions of its range were effectively protected. However, resource constraints and competing land‐use priorities often preclude this. Therefore, incorporating caribou into multiobjective assessments would provide an opportunity to enhance conservation planning efficiency (Martin et al., 2022; Wiersma & Sleep, 2018).

As a first step, an umbrella index can be valuable for identifying potential gaps in single‐species conservation efforts, comparing efficiency of alternative umbrella species, understanding under what conditions the value of an umbrella species could be maximized, and supporting protected area network planning (e.g., informing the single‐large‐or‐several‐small debate [Fahrig et al., 2022; May et al., 2019]). Regardless of whether one uses overlapping ranges, scenarios, or null and alternative models, careful evaluation of the assessment method for inferring anticipated conservation gains (i.e., umbrella value) is key to correctly interpreting findings and better informing land‐use planning.
